# T Cell Receptor Immune Repertoires Are Promptly Reconstituted After Methicillin-Resistant *Staphylococcus aureus* Infection

**DOI:** 10.3389/fmicb.2019.02012

**Published:** 2019-08-30

**Authors:** Jiangjun Liu, Zhongqiang Liu, Yuanqi Zhu, Bingzi Dong, Zheng Cai, Qing Liang, Kejia Wang

**Affiliations:** ^1^Department of Basic Medical Sciences, School of Medicine, Xiamen University, Xiamen, China; ^2^Department of Orthopedic Surgery, Qilu Hospital of Shandong University, Shandong, China; ^3^Department of Clinical Laboratory, The Affiliated Hospital of Qingdao University, Qingdao University, Qingdao, China; ^4^Department of Endocrinology and Metabolism, The Affiliated Hospital of Qingdao University, Qingdao University, Qingdao, China; ^5^Department of Pharmaceutics, Changhai Hospital, Second Military Medical University, Shanghai, China

**Keywords:** T cell receptor, immune repertoire, methicillin-resistant *Staphylococcus aureus*, next-generation sequencing, complementarity determining region 3

## Abstract

T cells represent a subset of lymphocytes characterized by immunosurveillance and immunoregulation function. Peripheral blood mononuclear cells (PBMCs) are enriched in T cells, which exert critical antimicrobial roles in infectious diseases. High-throughput sequencing of the T cell receptor (TCR) provides deep insight into monitoring the immune microenvironment. Flow cytometry was used to analyse the distribution of αβ/γδ T cells and their CD69, IFN-γ/IL-17 expression from PBMCs. Here, we utilized next-generation sequencing (NGS) to detect the complementarity determining region 3 (CDR3) of TCRβ (TRB) and TCRδ (TRD) chain after methicillin-resistant *Staphylococcus aureus* (MRSA) infection. Our data demonstrated a significant increase in the activation of αβ and γδ T cells after MRSA infection. Simultaneously, significantly high CDR3 amino acid (AA) diversity and markedly reconstituted TCR immune repertoires were observed after MRSA infection. Finally, we identified several MRSA-specific initial CDR3 AA motifs after MRSA infection. Our work reveals the profiles of TRB and TRD immune repertoires in response to MRSA and demonstrates a reconstitution of the TCR immune repertoire after MRSA infection.

## Introduction

T cells, which are mainly responsible for the cellular immune response, can be divided into two subsets, αβ and γδ, according to their T cell receptor (TCR) usage. The β and δ chains are composed of variable (V), diversity (D), joining (J), and constant (C) domains, in which the area that V-D-J joins (from the terminus of the V domain to the beginning of the J domain) is referred to as the CDR3 (Calis and Rosenberg, 2014). The specificity and diversity of αβ and γδ T cells depend on the CDR3 region of the TCR, which originates from the recombination of somatic V and J gene elements and random nucleotide excision and addition at the V-D and D-J junctions (Pannetier et al., 1993; Nikolich-Zugich et al., 2004). There are notable differences in the array of genetic elements, recombination and diversity, but the basic heterodimeric structure of αβ and γδ is similar (Girardi, 2006).

The majority of T cells in the peripheral blood expresse a TCR heterodimer consisting of α and β chains, which bind to CD3 to interact with the major histocompatibility complex (MHC) on antigen-presenting cells (APCs) to recognize antigens (Roche and Cresswell, 2016). Unlike most αβ T cells, γδ T cells recognize ligands that vary much in size, composition and molecular structure with or without MHC restriction, including MHC and non-MHC cell surface molecules, soluble proteins and smaller peptides, phospholipids, prenyl pyrophosphates and sulfatide (Davis and Bjorkman, 1988; Brandes et al., 2005; Vantourout and Hayday, 2013). γδ T cells mainly sense early environmental stimulus by indirect interactions to mediate immunosurveillance and immunoregulation (Born et al., 2013, 2017; Fleming et al., 2017). In addition, although there are few V and J elements that may be utilized for rearrangements, γδ T cells represent a minority of T cells that only act as a first defense to initiate local immunosurveillance (Gao et al., 2008). It is considered that TCR repertoires are closely related to individual immune status under certain circumstances, and TCR repertoires will change in response to stimuli (infection and disease) (Singhal et al., 2013; Hou et al., 2016; Poschke et al., 2016). However, the precise profile of TCR repertoires in infectious disease is still uncertain.

*Staphylococcus aureus*, considered a health care- and community-acquired pathogen, is responsible for a large amount of infectious diseases ranging from soft tissue infections to bacteremia, endocarditis, pneumonia and osteomyelitis (Lowy, 1998; Dayan et al., 2016). There is a global increase in the incidence of methicillin-resistant *Staphylococcus aureus* (MRSA) infection in both adults and children. According to US Centers for Disease Control, it is estimated that MRSA is responsible for 80,000 invasive infections and 11,000 infection-related deaths each year (Solomon and Oliver, 2014). Staphylococcal enterotoxins that originate from MRSA frequently cause septicemia or toxic shock syndrome. Staphylococcal enterotoxins are described as “superantigens” to stimulate T cell proliferation and activation, leading to the release of T cell-derived cytokines (Gjorloff et al., 1991; D’Orazio et al., 1995). Emerging studies indicate that key repertoire signatures of T cells evolve during infection and in response to vaccination (Linnemann et al., 2013; Pompano et al., 2014; Song et al., 2017). Nevertheless, the association of TCR repertoires and CDR3 diversity in immunosurveillance and immunoregulation has not been clarified. Therefore, the characterization of TCR immune repertoires may improve our basic understanding of T-cell immunology and help to identify optimal TCRs for immunotherapy.

Technical advances now offer the opportunity for monitoring the immune microenvironment by TCR next-generation sequencing (NGS). NGS is a potent tool to evaluate the clonal composition of TCR immune repertoires and to comprehensively probe the complex TCR diversity. Consequently, monitoring of TCR immune repertoires is appropriate for addressing fundamental questions of T cell immunology in MRSA infection. In this study, we measured the quantity and activity of αβ and γδ T cells in peripheral blood, and TCR NGS was used to monitor the expression pattern and distribution of the TRB and TRD repertoire in peripheral T cells, which is conducive to uncover an essential evolution of TRB and TRD immune repertoires during MRSA infection.

## Materials and Methods

### Ethics Statement

Animal experiments (QDU20180114) were approved by the Animal Care and Use Committee of the Qingdao University. All procedure meet the international criteria on humane treatment that spare the animal needless pain and suffering. All animals were housed in a pathogen-free state, at a temperature of 22 ± 1°C with 45 ± 10% humidity, and a 12 h light/12 h dark cycle. The mice health and behavior were monitor every 12 h and euthanized under moribund state (anorexia, immobile, and frizzy).

### Animals and MRSA Infection

All C57BL/6 mice (purchased from Daren Fortune Animal Technology Co., Ltd., Qingdao, China) were maintained in the SPF barrier facility animal rooms according to protocols approved by the Qingdao University of Medicine Institutional Animal Care and Use Committee. The MRSA strain (USA300) used in this study was obtained from ATCC and incubated with Bacto Tryptic Soy Broth (TSB) (21825, BD Biosciences, San Jose, CA, United States) liquid medium. For MRSA infection, mice were administered 1 × 10^7^ CFU by intraperitoneal injection in a total volume of 100 μl. Mice injected with 0.9% saline were used as controls. Twenty-four hours after injection, mice were sacrificed for peripheral blood mononuclear cell (PBMC) isolation.

### PBMC Isolation and FACS Analysis

Peripheral blood mononuclear cells were isolated from whole-blood specimens by using Ficoll (Solarbio Life Sciences, Beijing, China) density gradient centrifugation. Subsequently, the cell pellets were washed twice with PBS for further analysis. Isolated immunocytes were incubated with fluorescently conjugated antibodies directed against mouse CDR3, TCRβ and TCRγδ from BioLegend (San Diego, CA) for 20 min at 4°C in the dark. For cytokine detection, the selected cells were stained using antibodies against murine CD69, IFN-γ and IL-17 from BD Pharmingen (San Jose, CA, United States) for 20 min at 4°C in the dark after permeabilizing with permeabilization buffer. Stained cells were assessed on a BD FACSAria II (BD Biosciences, San Jose, CA, United States) and analyzed with FlowJo Version 10 Software (TreeStar, Ashland, OR, United States).

### RNA Extraction

The RNAprep Pure Cell/Bacteria Kit (TIANGEN Biotech, Beijing, China) was used for total RNA extraction according to the manufacturer’s specifications. The concentrations of RNA were quantified using an Eppendorf BioPhotometer Plus (Eppendorf, Hamburg, Germany). Reverse-transcription reactions were performed using a Transciptor First Strand cDNA Synthesis Kit (Roche Applied Science, Penzberg, Germany) according to the manufacturer’s protocol on a T100^TM^ Thermal Cycler (Bio-Rad Laboratories, Inc., Hercules, CA, United States).

### TCR Repertoire Amplification

For TCR CDR3 amplicon generation, two-round multiplex PCR was performed with a Multiplex PCR Assay Kit Ver. 2 (TaKaRa, Dalian, China) using specific primers designed for functional V and C gene segments of the mouse TCR chain according to the international Immunogenetics information system (IMGT)^1^ (Liang et al., 2018).

### Analysis of High-Throughput Sequencing Data

For the purification of PCR amplicons, PCR products were loaded on 1% TBE-agarose for gel electrophoresis (Supplementary Figure S1). Bands at 100–300 bp amplicons were excised and purified using the QIAquick Gel Extraction Kit (Qiagen). Illumina adaptor sequences were introduced to the PCR amplicons for further analysis using the Illumina HiSeq X Ten platform with a read length of 2 × 150 bp. Library preparation of PCR amplicons was performed based on the “denature and dilution guide” provided by Illumina.

### Data Processing

The original data obtained from the Illumina HiSeq X Ten platform were converted to raw reads by filtering the low-quality sequences. Paired-end V and J sequences of TRB and TRD were identified using BLAST Plus on IMGT/HighV-Quest by a standard algorithm (Alamyar et al., 2012). TCR diversity was evaluated by Gini coefficient, Shannon diversity and Rank-abundance, which have been widely used for assessing the richness and diversity of TCR as previously described (Birtel et al., 2015; Chen et al., 2016).

### Statistical Analysis

All statistical analyses were calculated with GraphPad Prism 6 Package (GraphPad Software, La Jolla, CA, United States) using paired Student’s *t*-test for repertoire comparisons. Data are presented as the mean ± standard deviation. A *P* < 0.05 was considered significant.

## Results

### MRSA Infection Activates αβ and γδ T Cells

**FIGURE 1:**
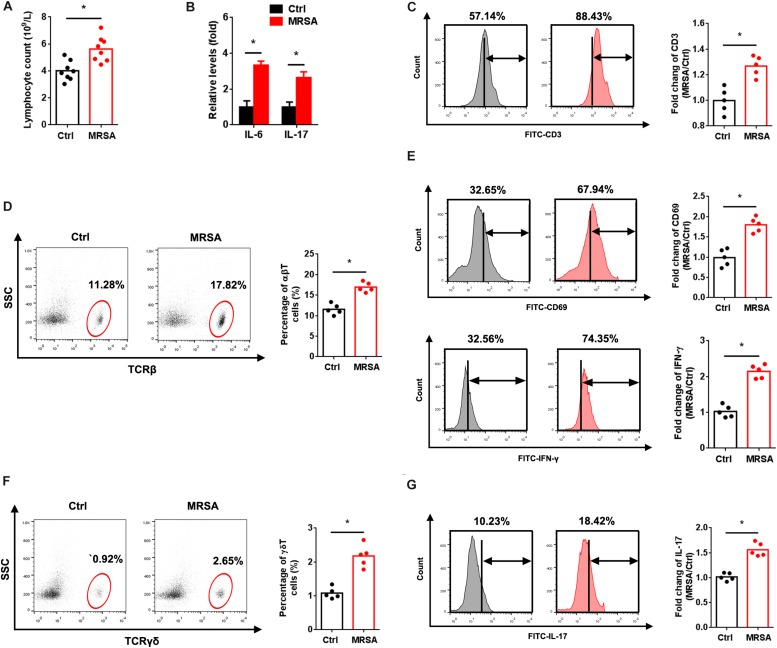
MRSA infection induces αβ and γδ T cell activation. Mice were administered 1 × 10^7^ CFU MRSA or 0.9% saline by intraperitoneal injection for 24 h. **(A)** Lymphocytes were measured from the peripheral blood (*n* = 8). **(B)** Serum IL-6 and IL-17 were detected by ELISA (*n* = 8). PBMCs isolated from peripheral blood were analyzed by FACS. **(C)** Fluorescence intensity of CD3 in the ctrl and MRSA groups (*n* = 5). Representative images for TCRβ **(D)** and TCRγδ **(F)** were taken after MRSA infection. αβ and γδ T cells harvested from PBMCs were gated and tested for expression of CD69, IFN-γ **(E)** and IL-17 **(G)** (*n* = 5). Experiments were performed using 5 or 8 mice per group. ^∗^*P* < 0.05.

To explore the immune status in response to MRSA strain USA300 *in vivo*, we measured the population of lymphocytes and levels of IL-6 and IL-17 in peripheral blood. As expected, a significant influx of lymphocytes along with increased IL-6 and IL-17 levels were observed with MRSA infection (Figures 1A,B). FACS analysis also revealed elevated total (Figure 1C), αβ (Figure 1D), and γδ (Figure 1F) T cells in the peripheral blood of MRSA-infected mice. To determine whether USA300 activates αβ and γδ T cells, we examined the *in vivo* expression of CD69, IFN-γ and IL-17 in T cells. There were obvious increases in expression of CD69 and IFN-γ in αβ T cells (Figure 1E) and IL-17 in γδ T cells (Figure 1G). A significant increase in the activation of αβ and γδ T cells was also observed.

### Profiling of Peripheral TCR Sequencing

Next, an NGS approach based on multiplex PCR of cDNA from peripheral T cells was used to detect TCR immune repertoires. For TCR identification, paired-end V and J region sequences were identified using BLAST Plus on IMGT/HighV-Quest by a standard algorithm (Alamyar et al., 2012). This yielded 5.59 × 10^6^ to 9.38 × 10^6^ productively TRB blast reads per sample and 4.88 × 10^6^ to 7.19 × 10^6^ productively TRD blast reads per sample. The matching rate was 82.96–96.42% in the TRB Blast and 73.85–89.96% in the TRD Blast. The total number of TRB CDR3 reads was 5.26 × 10^6^ to 9.07 × 10^6^, with an average of 6.52 × 10^4^ CDR3 amino acid (AA) clones per sample. In contrast, the total number of TRD CDR3 reads was 3.74 × 10^6^ to 6.74 × 10^6^, with an average of 4.90 × 10^4^ CDR3 AA clones per sample (Supplementary Table S1).

### The Usage Patterns of V, D, and J Gene Segments After MRSA Infection

**FIGURE 2:**
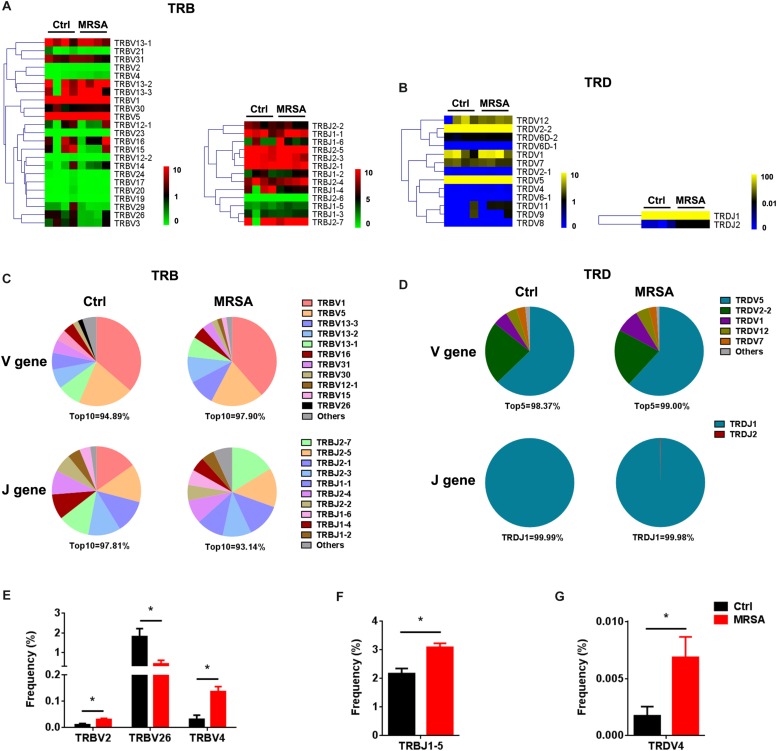
The usage patterns of V, D, and J gene segments after MRSA infection. Heatmaps of hierarchical clustering of V and J gene frequencies for TRB **(A)** and TRD **(B)**. **(C,D)** The distribution of the V and J gene segments. **(E–G)** The frequency of V and J gene segment usage that had significant differences between the ctrl group and the MRSA group. Experiments were performed using 4 mice per group. ^∗^*P* < 0.05.

We identified a total of 36 distinct V gene segments (TRB, 23; TRD, 13) and 15 distinct J gene segments (TRB, 13; TRD, 2) from all samples (Supplementary Table S2). Generally, the usage pattern and frequency of most V and J gene segments (in both TRB and TRD) were similar between the ctrl group and the MRSA group (Figures 2A,B). The most frequent V gene segments were TRBV1 (36.34% in the ctrl group, 38.55% in the MRSA group) and TRDV5 (62.80% in the ctrl group, 61.84% in the MRSA group). The most frequent J gene segments were TRBJ2-1 (14.74%) in the ctrl group and TRBJ2-7 (16.31%) in the MRSA group. TRDJ1 accounted for almost all of the TRDJ in both the ctrl and MRSA groups (Figures 2C,D). However, these differences were significant in the frequency of TRBV2, TRBV26, TRBV4, TRBJ1-5, and TRDV4 (Figures 2E–G).

**FIGURE 3:**
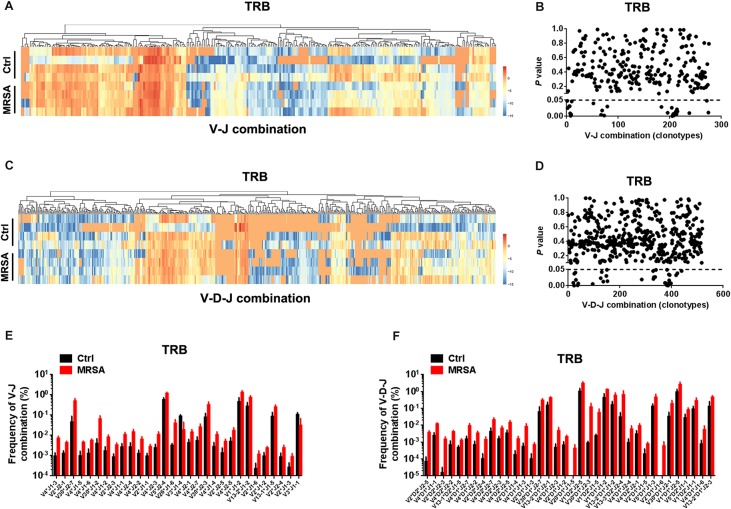
The usage patterns of V-J and V-D-J combination for TRB after MRSA infection. Heatmaps showing the hierarchical clustering of differentially expressed percentage of V-J **(A)** and V-D-J **(C)** combinations for TRB. Comparison of V-J **(B)** and V-D-J **(D)** combinations usage between the ctrl group and the MRSA group. Every dot indicates a *P*-value of comparison of V-J or V-D-J clonotype usage between two groups (dotted line refers to *P* = 0.05). The frequency of V-J **(E)** and V-D-J **(F)** combinations that had statistical difference between the ctrl group and the MRSA group was shown. Experiments were performed using 4 mice per group.

We also detected the composition of V-J gene combinations and V-D-J combinations. A total of 298 distinct V-J gene combinations (TRB, 276; TRD, 22) and 541 distinct V-D-J gene combinations (TRB, 519; TRD, 22) were identified from all samples (Supplementary Table S3). Heatmaps were generated according to the usage frequency of V-J combinations and V-D-J combinations for TRB (Figures 3A–C). Strikingly, compared to the ctrl group, there were 31 V-J combinations (TRB, 30; TRD, 1) and 37 V-D-J combinations (TRB, 36; TRD, 1) that exhibited significantly altered usage in the MRSA group (Figures 3B–D and Supplementary Figure S2A). Interestingly, the frequency of a majority of V-J and V-D-J combinations was enhanced in the MRSA group, compared to the ctrl group (Figures 3E,F and Supplementary Figure S2B).

### An Abundant Diversity of TCR Repertoires After MRSA Infection

**FIGURE 4:**
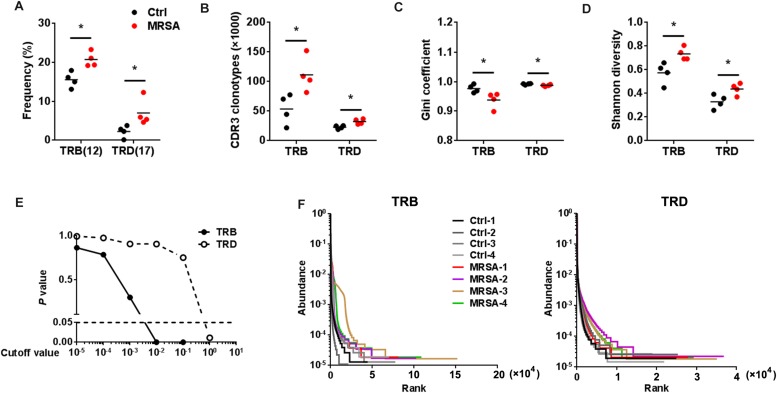
The diversity of TCR repertoires diversity increased after MRSA infection. **(A)** The frequency of the CDR3 AA length = 12 for TRB and the CDR3 AA length = 17 for TRD. Comparison of the TCR repertoire diversity by CDR3 clonotypes **(B)**, Gini coefficient **(C)**, and Shannon index **(D)**. **(E)** Comparison of all clone frequencies for TRB and TRD between the ctrl group and the MRSA group. The cutoff value depends on the clonal frequency exceeding a certain threshold. **(F)** Rank-abundance analysis of the TRB and TRD repertoires from all samples. Experiments were performed using 4 mice per group. ^∗^*P* < 0.05.

The types of CDR3 AA clones determine the diversity of the TCR repertoires. In the current study, 583,354 distinct TRB CDR3 AA clones and 205,817 distinct TRD CDR3 AA clones (Supplementary Table S4) were identified. There were no significant differences observed in length tendency in CDR3 AA length after MRSA treatment (Supplementary Figure S3), and only the frequency of 12 AA length for TRB CDR3 and the 17 AA length for TRD CDR3 were different after MRSA infection (Figure 4A). Although the total TCR CDR3 AA clones were distinguishing among different mice, MRSA infection, as expected, led to clear increases in CDR3 AA clonotypes, including TRB and TRD (Figure 4B). Furthermore, Gini coefficient and Shannon diversity were used to compare CDR3 AA diversity (Figures 4C,D). All results suggested markedly enlarged TRB and TRD CDR3 AA diversity and a change in CDR3 AA usage (Figure 4E) after MRSA infection. Rank-abundance analysis revealed a shrunken TCR richness and evenness during MRSA infection (Figure 4F).

### Resetting of the TCR Repertoires After MRSA Infection

**FIGURE 5:**
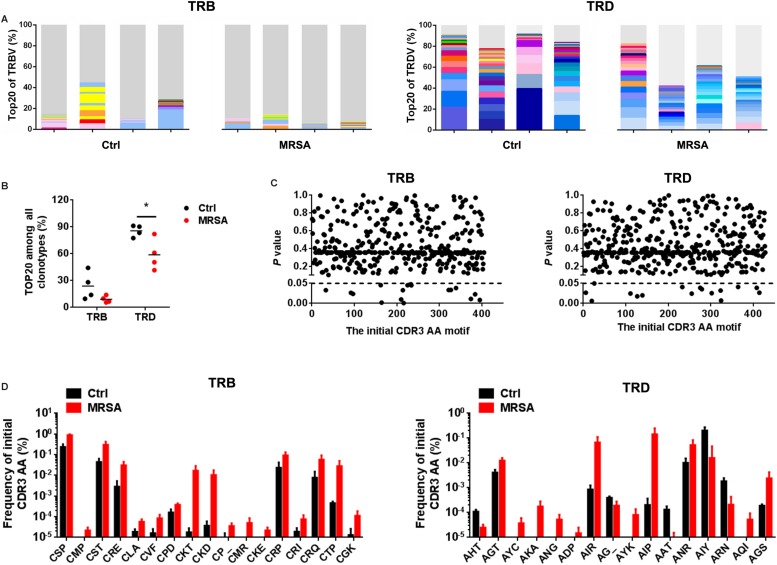
Reconstitution of various TCR repertoires after MRSA infection. **(A)** Fraction of the identified Top20 TRB clones (left) and Top20 TRD clones (right). Colors indicate Top20 clones (each color represents a clone); grays represent non-Top20 clones. **(B)** Frequency of the Top20 clones among all clones for TRB and TRD. **(C)** Comparison of the fraction of the initial CDR3 AA motif between the ctrl group and the MRSA group. **(D)** The frequency of the initial CDR3 AA motif that had significant differences between the ctrl group and the MRSA group. Experiments were performed using 4 mice per group. ^∗^*P* < 0.05.

To investigate the reconstitution of TCR repertoires, we evaluated V-CDR3-J usage during MRSA infection (Supplementary Table S5). Overall, TRD repertoires were dominated by high-frequency CDR3 AA clones, which the Top20 clones accounting for 58.82% ± 17.38% in the ctrl group and 85.66% ± 6.42% in the MRSA group. In contrast, the Top20 clones of TRB only accounted for 15.19% ± 8.58% in the ctrl group and 18.86% ± 7.85% in the MRSA group (Figure 5A). Notably, the usage of both TRD Top20 clones and TRB Top20 clones was shrunken in samples after MRSA infection (Figure 5B). In addition, comparison of CDR3 AAs showed that MRSA induced a changed usage of the initial CDR3 AA motif (Supplementary Table S6 and Figures 5C,D). Together, these results revealed that the TCR repertoires after MRSA infection were fundamentally different from those uninfected controls due to TCR rearrangements.

## Discussion

For decades, despite a profound understanding of T cell immunosurveillance and immunoregulation, much less is known about the regulatory mechanism of T cells in distinct settings (Girardi, 2006). Although preserved in various organs and mucosae, large differences in the relative frequency and composition indicate diverse function of T cell subsets between tissues (Gao et al., 2008; Born et al., 2017). In the peripheral blood, T cells have been shown to accumulate upon various infectious settings that result from exogenous pathogens. TCRs specific for antigens have important roles in the host defense against infections. Accordingly, understanding the TCR profile in certain conditions may provide an effective approach to monitor the immune system and control infection.

Accumulating studies indicate that MRSA triggers a systemic immune response, contributing to tissue damage as a result of dysregulated T cell activation and excessive cytokine production (Yoh et al., 2000; Parker et al., 2015; Lee et al., 2016). In this course, the T cell itself provides exact identification synergistically with both parenchymal and non-parenchymal cells to promote pathogenesis. Nevertheless, the rearrangement of TCR immune repertoires in the process of MRSA infection is still uncertain. In preceding studies, MRSA induces the proliferation of resting T cells, as manifested by massive T cell activation and the release of T cell-derived cytokines (Yoh et al., 2000; Chan et al., 2015). Consistent with a previous study, our results revealed enhanced activation of αβ and γδ T cells in the peripheral blood after MRSA infection, demonstrating the crucial role of αβ and γδ T cells after MRSA infection.

In addition, our work reveal an intact TCR profile of V, D, and J gene elements and CDR3 AA clonotypes after MRSA infection. In the current study, a more diverse TRB repertoire was found in individuals, whereas the TRD repertoire exhibited a centralized distribution with chaotic CDR3 AA length. These data were consistent with previous studies that indicated that TRB has a highly variable CDR3 length (Girardi, 2006). In addition, notable increased TCR diversity and altered TCR repertoires were observed after MRSA infection. Our observation of the transformation of TCR immune repertoires both in αβT and γδT cells after MRSA infection provides evidence of MRSA-associated immune repertoires in the adaptive immune system. Indeed, these results indicate that the transformation of TCR immune repertoires is indispensable for antigen recognition in the adaptive immune system after MRSA infection.

Until now, the molecular mechanism of T cell immunity has rarely been investigated in MRSA infection. In the past, more attention has focused on a single parameter of the immune system in response to pathogens. Identifying and tracking TCR immune repertoires by NGS provides a novel strategy to understand the dynamics and distribution of TCR genes in MRSA infection instead of trying to correlate single parameters with the complex process. Multi-parameter analysis of MRSA-responsive TCR represents a “footprint” of immune conditions. Interestingly, our ongoing study shows that the highly aberrant inflammatory milieu is associated with TCR reconstitution. Thus, it is postulated that TCR acts more as a “commander” than as an “executor” to control the immune microenvironment after MRSA infection. However, the mechanism of TCR reconstitution in T cell activation and other immunocyte subset recruitment are still not clear, and how TCR immune repertoires modulate the inflammatory milieu needs to be further examined.

## Conclusion

Our study exhibits the profile of TCR immune repertoires after MRSA infection. Through tracking the kinetics of the reconstitution of TCR by NGS, our work has delivered the message that the quick reconstitution of TCR immune repertoires after MRSA infection is essential for T cell immunity. Our work provides novel insights to identify signatures of infectious diseases and accelerates discoveries of vaccine design and immunotherapy.

## Data Availability

All datasets generated for this study are included in the manuscript and/or the Supplementary Files.

## Ethics Statement

Animal experiments (QDU20180114) were approved by the Animal Care and Use Committee of the Qingdao University. All procedure meet the international criteria on humane treatment that spare the animal needless pain and suffering. All animals were housed in a pathogen-free state, at a temperature of 22 ± 1°C with 45 ± 10% humidity, and a 12 h light/12 h dark cycle. The mice health and behavior were monitor every 12 h and euthanized under moribund state (anorexia, immobile, and frizzy).

## Author Contributions

KW contributed to the study conception and design. JL, ZL, YZ, BD, and QL performed the experiments. JL and ZC analyzed the data. All authors contributed to the manuscript drafting and approved the final version of the manuscript.

## Conflict of Interest Statement

The authors declare that the research was conducted in the absence of any commercial or financial relationships that could be construed as a potential conflict of interest.

## Footnotes

1 http://www.imgt.org/

## Supplementary Material

FIGURE S1 Two-round nested amplicon arm-PCR for TRB and TRD between ctrl group and MRSA group.

FIGURE S2 The usage patterns of V-J and V-D-J combination for TRD after MRSA infection. **(A)** The comparison of V-J and V-D-J combinations between ctrl group and MRSA group (dot line refer to P=0.05). **(B)** The frequency of V4-J2 and V12-D2-J1 between ctrl group and MRSA group. Experiments were performed using 4 mice per group. ^∗^*P* < 0.05.

FIGURE S3 The distribution of CDR3 AA length in the TRB **(left)** and TRD **(right)** between ctrl group and MRSA group.

TABLE S1 The analysis of TCRβ immune repertoire sequencing data.

TABLE S2 The distribution of V and J gene segments in each sample.

TABLE S3 The distribution of V-J and V-D-J combinations in each sample.

TABLE S4 The distribution of CDR3 AA clonotypes in each sample.

TABLE S5 The distribution of V-CDR3-J combinations in each sample.

TABLE S6 The distribution of initial CDR3 AA motifs in each sample.
