# Supplementary material for: T Cell Receptor Immune Repertoires Are Promptly Reconstituted After Methicillin-Resistant Staphylococcus aureus Infection
Source: Front Microbiol. 2019 Aug 30;10:2012. doi: 10.3389/fmicb.2019.02012 (PMC6730595; doi:10.3389/fmicb.2019.02012)
Supplement: FIGURE S1 — Two-round nested amplicon arm-PCR for TRB and TRD between ctrl group and MRSA group. [file Data_Sheet_1.PDF]

T cell receptor immune repertoires are promptly reconstituted after methicillin-resistant Staphylococcus aureus infection

Running title: TCR immune repertoires in MRSA infection

Jiangjun Liu[1, 2], Zhongqiang Liu[3], Yuanqi Zhu[3], Kang Sun[2], Qing Liang[1], Ping Zhu[4], Kejia Wang[1*]

[1] Department of Basic Medical Sciences, School of Medicine, Xiamen University, Xiamen, Fujian, China

[2] Department of Orthopaedics, The Affiliated Hospital of Qingdao University, Qingdao University, Qingdao, Shandong, China

[3] Department of Clinical Laboratory, The Affiliated Hospital of Qingdao University, Qingdao University, Qingdao, Shandong, China

[4] Department of Obstetrics and Gynecology, No. 971 Navy Hospital of PLA, Qingdao, Shandong, China

[*]Correspondence: Kejia Wang, Department of Basic Medical Sciences, School of Medicine, Xiamen University, Xiamen, Fujian, China. E-mail: wangkejia@xmu.edu.cn

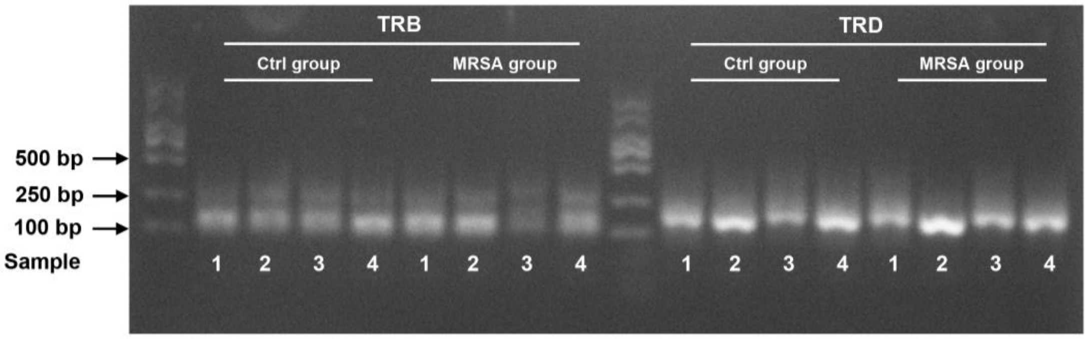

Supplementary Figure 1 Two-round nested amplicon arm-PCR for TRB and TRD between ctrl group and MRSA group.

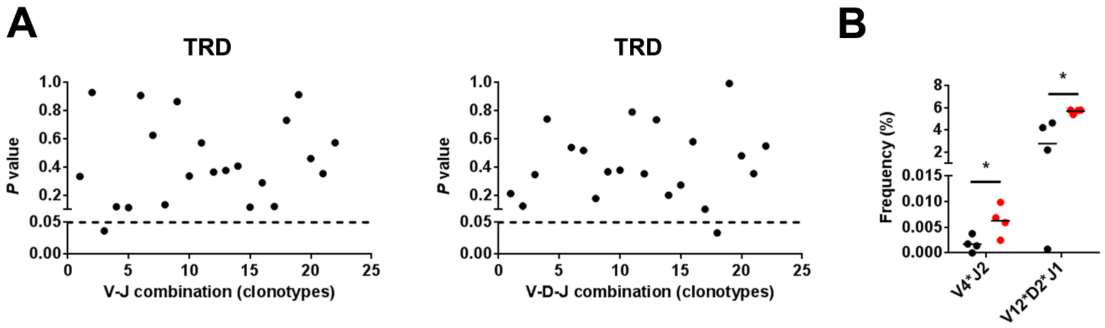

Supplementary Figure 2 The usage patterns of V-J and V-D-J combination for TRD after MRSA infection. (A) The comparison of V-J and V-D-J combinations between ctrl group and MRSA group (dot line refer to P=0.05). (B) The frequency of V4-J2 and V12-D2-J1 between ctrl group and MRSA group. Experiments were performed using 4 mice per group. *, P<0.05.

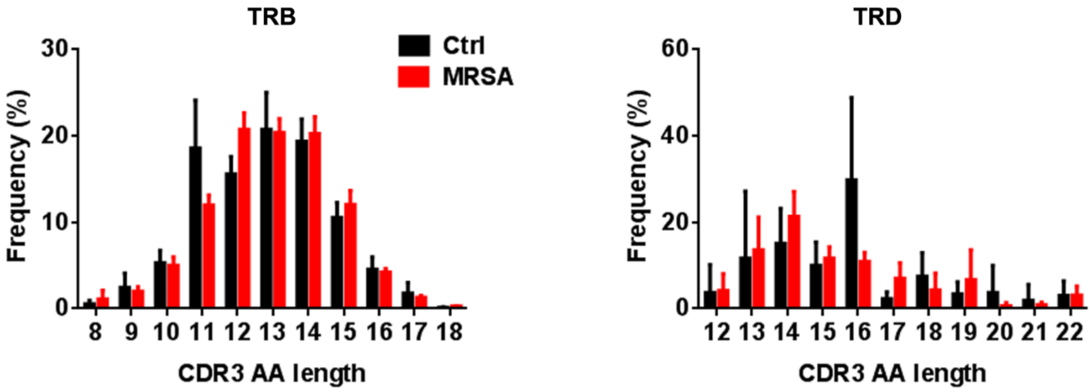

Supplementary Figure 3 The distribution of CDR3 AA length in the TRB (left) and TRD (right) between ctrl group and MRSA group.
